# A Phase II trial of axitinib in patients with various histologic subtypes of advanced thyroid cancer: long-term outcomes and pharmacokinetic/pharmacodynamic analyses

**DOI:** 10.1007/s00280-014-2604-8

**Published:** 2014-10-15

**Authors:** E. E. W. Cohen, M. Tortorici, S. Kim, A. Ingrosso, Y. K. Pithavala, P. Bycott

**Affiliations:** 1 Division of Biological Sciences, University of Chicago, Chicago, IL USA; 2 Pfizer Global Research and Development, Pfizer Inc., Collegeville, PA USA; 3 Clinical Development, Pfizer Oncology, San Diego, CA USA; 4 Clinical Development, Pfizer Oncology, Milan, Italy; 5 Clinical Pharmacology, Pfizer Oncology, San Diego, CA USA; 6 Present Address: Department of Medicine, University of California San Diego Moores Cancer Center, 3855 Health Sciences Drive, La Jolla, CA 92093 USA

**Keywords:** Axitinib, Iodine-refractory, Pharmacokinetic, Pharmacodynamic, Thyroid cancer

## Abstract

**Purpose:**

Axitinib, a potent and selective second-generation inhibitor of vascular endothelial growth factor receptors, has shown activity in advanced thyroid cancer in a Phase II study. We report updated overall survival and pharmacokinetic/pharmacodynamic (PK/PD) analyses from the study.

**Methods:**

Patients (*N* = 60) with advanced thyroid cancer of any histology for whom iodine-131 (^131^I) failed to control the disease or ^131^I was not appropriate therapy were administered axitinib 5 mg twice daily. Objective response rate (primary endpoint), duration of response, progression-free survival, overall survival, safety, and PK/PD relationships were assessed.

**Results:**

Objective response rate was 38 % [23 partial responses; 95 % confidence interval (CI) 26–52], and 18 (30 %) patients had stable disease lasting ≥16 weeks. Responses occurred in all histologic subtypes. With median follow-up of 34 months (95 % CI 32–37), median overall survival was 35 months (95 % CI 19–not estimable), median progression-free survival was 15 months (95 % CI 10–20), and median duration of response was 21 months (95 % CI 13–46). Most common Grade 3/4 treatment-related adverse events included hypertension (13 %), proteinuria (8 %), diarrhea (7 %), weight decrease (7 %), and fatigue (5 %). PK/PD analyses revealed trends toward greater tumor size reduction and response probability with higher axitinib plasma exposures.

**Conclusions:**

Axitinib appears active and well tolerated in patients with various histologic subtypes of advanced thyroid cancer, demonstrating durable responses and long overall survival. Axitinib may be useful for the treatment of advanced thyroid cancer.

**Electronic supplementary material:**

The online version of this article (doi:10.1007/s00280-014-2604-8) contains supplementary material, which is available to authorized users.

## Introduction

Thyroid cancer was diagnosed in approximately 212,000 individuals worldwide and resulted in about 35,000 deaths in 2008 [1]. Its incidence has increased, on average, by 58 % in most populations [2]. Despite its rising incidence, thyroid cancer mortality in the European Union has declined [3]. Survival is stage-dependent, with a 5-year relative survival rate of 57.3 % for distant disease [4].

Thyroid tumors have elevated levels of vascular endothelial growth factor (VEGF) compared with normal thyroid tissue [5], suggesting the VEGF pathway as an appropriate therapeutic target. Several tyrosine kinase inhibitors (TKIs) targeting the VEGF pathway (e.g., sorafenib [6–11], sunitinib [12–14], axitinib [15, 16], vandetanib [17, 18], pazopanib [19], motesanib [20, 21], cabozantinib [22–24], and lenvatinib [25]) have been evaluated in patients with advanced thyroid cancer.

Axitinib, a potent and selective second-generation inhibitor of VEGF receptors (VEGFRs) [26], is approved in the USA, European Union, and elsewhere for the treatment of advanced renal cell carcinoma (RCC) after failure of prior systemic therapy [27]. The activity of axitinib was previously reported in a Phase II trial of patients with various histologic subtypes of advanced thyroid cancer in whom iodine-131 (^131^I) failed to control the disease or ^131^I was not appropriate therapy [16]. The final clinical results with long-term outcomes, including updated overall survival (OS), and pharmacokinetic/pharmacodynamic (PK/PD) analyses from this trial are reported here.

## Materials and methods

### Study design

The primary objective of this Phase II study in patients with advanced thyroid cancer was to determine the activity of axitinib as measured by investigator-assessed overall objective response rate (ORR) per Response Evaluation Criteria in Solid Tumors (RECIST, v. 1.0) [28]. Complete response (CR) or partial response (PR) required confirmation at least 4 weeks after the first observation. The secondary objectives were to determine OS, progression-free survival (PFS), duration of response, and safety; obtain blood samples for population PK analyses; and explore relationships between clinical response and plasma-soluble proteins (i.e., VEGF and soluble VEGFR2 [sVEGFR2]).

This study was conducted in accordance with the Declaration of Helsinki, International Conference on Harmonization Guideline for Good Clinical Practice, study protocol, and all applicable local regulatory requirements and laws. Each participant provided written informed consent prior to inclusion in the study and agreed to comply with the study protocol. Study protocol and informed consent forms were approved by an institutional review board or independent ethics committee. The trial is registered on ClinicalTrials.gov (NCT00094055).

### Patients and assessments

Key patient inclusion/exclusion criteria and assessments were previously described [16]. Briefly, the trial enrolled adults with advanced thyroid cancer of any histology for whom ^131^I failed to control the disease or ^131^I was not appropriate therapy. Patients had Eastern Cooperative Oncology Group performance status 0 or 1, and at least one RECIST-defined target lesion not previously externally irradiated. Patients with uncontrolled hypertension [i.e., baseline blood pressure (BP) >140/90 mm Hg] were ineligible; antihypertensive medications were permitted. Prior treatment with antiangiogenic agents was not permitted.

### Treatments

Axitinib was administered orally at a starting dose of 5 mg twice daily without food or drink, other than water, for 2 h before and after each dose. Patients tolerating axitinib without treatment-related adverse events (AEs) Grade >1 according to the Common Terminology Criteria for Adverse Events (CTCAE, v3.0) [29] for any 8-week period were permitted a 20 % dose increase, unless responding to therapy. Patients developing subjectively intolerable, treatment-related Grade 2 AEs (except alopecia) uncontrolled by supportive treatment had axitinib interrupted and restarted at the same dose after resolution to Grade ≤1 or baseline. If resolution did not occur within 4 weeks, axitinib was discontinued.

Patients developing treatment-related Grade 3/4 non-hematologic AEs (except for alopecia) or treatment-related Grade 4 hematologic AEs uncontrolled by supportive treatment had axitinib interrupted. Upon adequate recovery to Grade ≤1 or baseline, treatment was resumed at a 20 % lower dose. If resolution did not occur within 4 weeks, axitinib was discontinued. Patients with recurring subjectively intolerable toxicity resumed axitinib at a 20 % lower dose upon adequate recovery. The previous report [16] specified a slightly different dose-modification schema that is more consistent with the axitinib prescribing information [27]. Axitinib was continued until disease progression, unacceptable toxicity, or consent withdrawal. Subsequent therapy was at the investigator’s discretion.

### Plasma pharmacokinetic samples and analysis

Samples (7 mL of whole blood) for population PK analysis were collected 15 min before and 1–2 h after the morning dose of axitinib (taken in clinic) on days 1 and 29 and every 8 weeks thereafter. Patients were required to take axitinib uninterrupted for ≥3 days before PK blood sample collection (not applicable on day 1).

Axitinib plasma concentrations were measured using validated high-performance liquid chromatography with tandem mass spectrometric detection (Charles River Laboratory Preclinical Services, Shrewsbury, MA, USA) [30–32]. Following population PK analysis, individual post hoc area under the plasma concentration–time curve at steady state (AUC_ss_) was calculated as follows:

$${\text{AUC}}_{\text{ss-study}} = {\text{average}}\;{\text{total}}\;{\text{daily}}\;{\text{dose}}\;{\text{during}}\;{\text{entire}}\;{\text{time}}\;{\text{on}}\;{\text{study}}/{\text{CL}}$$

and

$${\text{AUC}}_{{{\text{ss-cycle}}1}} = {\text{average}}\;{\text{total}}\;{\text{daily}}\;{\text{dose}}\;{\text{during}}\;{\text{cycle}}\;1/{\text{CL}}$$

where CL is systemic plasma clearance for axitinib (individual post hoc clearance estimated from population PK analysis), AUC_ss-study_ is the average AUC_ss_ across entire time on study, and AUC_ss-cycle1_ is the average AUC_ss_ during cycle 1.

### Plasma-soluble protein biomarkers

Plasma samples for measurement of VEGF and sVEGFR2 levels were collected on day 1 and every 8 weeks thereafter. Details of the bioanalytical methodology for measurement of plasma-soluble proteins were previously described [16].

### Statistical analysis

Sample size was based on a two-stage Simon minimax design [33] to evaluate the null hypothesis that the true ORR with axitinib was ≤5 % and the alternative hypothesis that ORR was ≥20 %, with type I (α) and type II (β) error rates of 0.10. Target accrual was 18 patients in stage I, with 14 additional patients in stage II if one or more confirmed responses were observed. Twenty-eight additional patients (total of 60) were treated to gain additional safety and activity information. Response rate was summarized, and confidence interval (CI) calculated using a method based on binomial distribution. Kaplan–Meier methods were used to estimate the median PFS, duration of response, and OS; corresponding CIs were calculated. Thirteen patients continued axitinib in an ongoing extension, study A4061008 (ClinicalTrials.gov, NCT00828919), in which only safety data were collected. Efficacy and safety data reported here for those 13 patients were based on data collected from this original trial.

Initial relationships between axitinib exposure and change in tumor size, as measured by sum of longest diameter (SLD) of target lesions, were explored using a simple linear regression analysis. Patients were stratified by axitinib AUC_ss_ (higher or lower than population median), and change from baseline in soluble proteins was compared using descriptive statistics. Patients were grouped into quartiles according to change from baseline in soluble proteins, and the proportion of RECIST responders (± standard deviation) was assessed.

Logistic regression for probability of PR was performed. The probability of achieving PR was assessed as a function of AUC_ss-cycle1_. Odds ratio per 1 ng h/mL change in AUC was calculated as:

$${\text{odds}}\;{\text{ratio}} = \exp^{\beta }$$

where* β* was the logistic regression slope coefficient.

## Results

### Patients and treatment

Baseline characteristics and demographics for the 60 patients enrolled were previously reported [16]. Briefly, median age was 59 years (range 26–84) and 78 % of patients were white; histologic subtypes are shown in Table 1. The majority (97 %) of patients received prior treatment; however, patients had not received prior antiangiogenic agents. All patients discontinued the study: 20 experienced insufficient clinical response; 14 planned to enroll in the ongoing extension study A4061008 (ClinicalTrials.gov, NCT00828919); 11 experienced non-fatal AEs; nine refused further participation; four died due to cardiorespiratory arrest, multiorgan failure, respiratory failure, or pneumonia; and two were lost to follow-up. One patient planning to enroll in the extension study had an optical malignancy and did not participate.

**Table 1:** Investigator-assessed objective response to axitinib: overall and by histologic subtype

	Complete response	Partial response	Stable disease^a^	Progressive disease	Indeterminate	Missing
	*n* (%)	*n* (%)	*n* (%)	*n* (%)	*n* (%)	*n* (%)
All patients (*N* = 60)	0	23 (38)^b^	18 (30)	5 (8)	9 (15)	5 (8)
Histologic subtype						
Papillary (*n* = 30)	0	10 (33)	10 (33)	3 (10)	4 (13)	3 (10)
Follicular (*n* = 15)^c^	0	9 (60)	4 (27)	1 (7)	1 (7)	0
Medullary (*n* = 11)	0	2 (18)	3 (27)	0	4 (36)	2 (18)
Anaplastic (*n* = 2)	0	1 (50)	0	1 (50)	0	0
Other (*n* = 2)	0	1 (50)^d^	1 (50)^e^	0	0	0

^a^Lasting ≥16 weeks. Of the 18 patients with stable disease ≥16 weeks, 17 had stable disease ≥30 weeks

^b^95 % confidence interval 26–52

^c^11 patients had the Hürthle cell variant

^d^Insular

^e^Neuroendocrine

Median duration of axitinib exposure was 11 months (range 0.2–47); 30 patients remained on treatment for at least 1 year. Median total daily dose of axitinib was 9 mg (range 2–15 mg). The axitinib dose was increased (>5 mg twice daily) in 24 patients eligible for dose titration, half of whom had a subsequent dose reduction. Another 24 patients had dose reductions.

### Clinical activity

Response assessments were unavailable for 14 patients because of missing post-baseline scans or indeterminate results (i.e., availability of only one or two post-baseline scans, which did not allow confirmation of either PR or duration of stable disease ≥16 weeks). For purposes of calculating ORR, they were considered non-responders. The investigator-assessed overall ORR was 38 % (95 % CI 26–52); no patient had CR, 23 had PR, and 18 (30 %) had stable disease lasting ≥16 weeks. Of the 18 patients with stable disease, 17 had stable disease lasting ≥30 weeks. Compared with our initial ORR analysis [16], five additional patients had confirmed PR with long-term follow-up. One patient with PR on day 600 had 22 % reduction in tumor size beginning on day 300, and another patient with PR on day 835 had 12 % reduction in tumor size beginning on day 166 that increased to 29 % on day 499. Responses occurred in all histologic subtypes (Table 1).

**Fig. 1:**
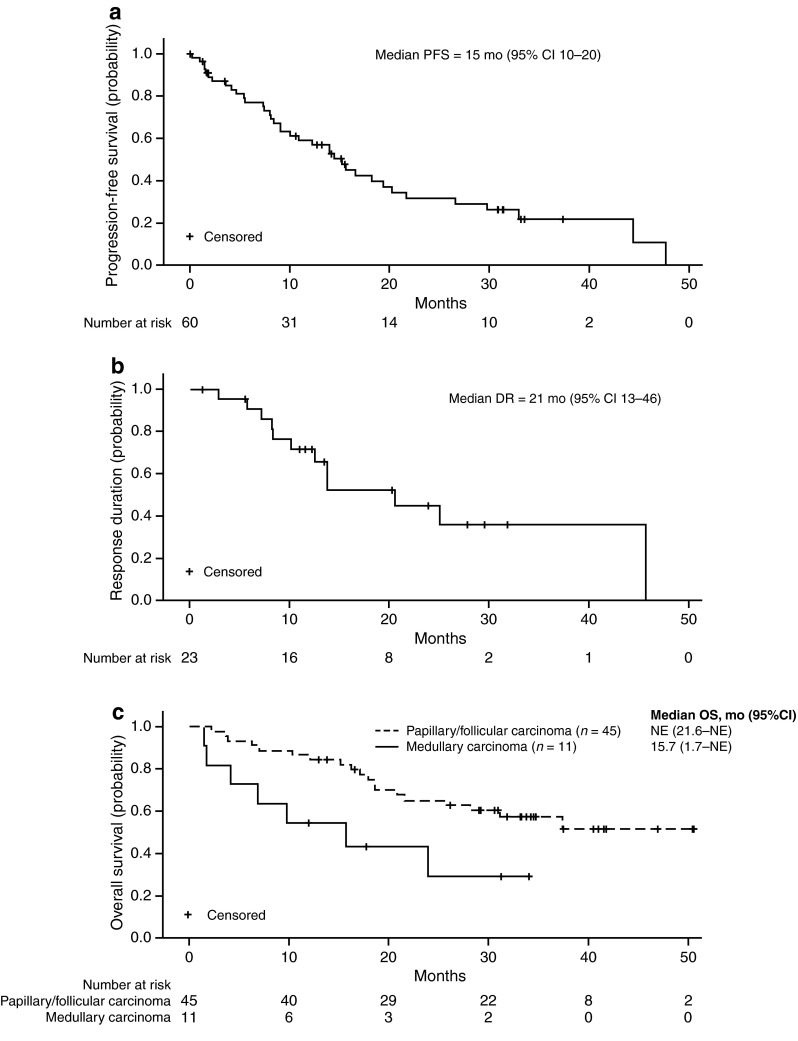
Kaplan–Meier curves for **a** investigator-assessed PFS; **b** investigator-assessed duration of response (DR) among responders; and **c** overall survival (OS) stratified by histologic subtype. Data for PFS, DR, and OS were not collected in the axitinib extension study; therefore, results for the 13 patients who rolled over to the extension study were based on data collected from the original trial. *CI* confidence interval, *NE* non-estimable

With an estimated median follow-up for survival of 34 months (95 % CI 32–37), disease progression or death had occurred in 37 (62 %) patients. These updated results, accounting for additional follow-up, are based on the final locked and clean database. These data were preliminarily reported [16], based on an active database and shorter follow-up of 16.6 months (95 % CI 15.0–21.2). In the final analysis, investigator-assessed median PFS was 15 months (95 % CI 10–20; Fig. 1a), median duration of response was 21 months (95 % CI 13–46; Fig. 1b), and median OS was 35 months (95 % CI 19 months–not estimable). Figure 1c illustrates OS according to histology.

### Safety

All-grade, treatment-related AEs (Table 2) occurred in 56 (93 %) patients; the most frequently reported AEs included diarrhea (63 %), fatigue (55 %), nausea (45 %), and hypertension (42 %). Grade 3/4 treatment-related AEs occurred in 21 (35 %) patients; the most common were hypertension (13 %), proteinuria (8 %), diarrhea (7 %), weight decrease (7 %), and fatigue (5 %). Five (8 %) patients experienced a total of six treatment-related Grade 4 AEs: reversible posterior leukoencephalopathy syndrome (RPLS) and hypertension (*n* = 1), proteinuria (*n* = 2), cerebrovascular accident (*n* = 1), and airway obstruction (*n* = 1). No Grade 5 (fatal) treatment-related AE was reported. Eight patients experienced 18 treatment-related serious AEs (SAEs): RPLS, mental status changes, and hypertension (*n* = 1); cerebrovascular accident and headache (*n* = 1); abdominal pain (*n* = 1); hypertension (*n* = 1); conduction disorder, diarrhea, nausea, vomiting, chest pain, and dehydration (*n* = 1); atrial fibrillation (*n* = 1); granuloma, airway obstruction, and respiratory tract hemorrhage (*n* = 1); and weakness (*n* = 1). Hypertension (*n* = 2) was the only treatment-related SAE experienced by more than one patient.

**Table 2:** Most common treatment-related adverse events

Adverse event^a^	All grades	Grade 3/4
	*n* (%)	*n* (%)
Any treatment-related AE	56 (93)	21 (35)
Diarrhea	38 (63)	4 (7)
Fatigue	33 (55)	3 (5)
Nausea	27 (45)	1 (2)
Hypertension	25 (42)	8 (13)
Weight decrease	19 (32)	4 (7)
Anorexia	18 (30)	0
Stomatitis	18 (30)	0
Dyspepsia	17 (28)	0
Headache	17 (28)	2 (3)
Mucosal inflammation	15 (25)	0
Proteinuria	15 (25)	5 (8)
Palmar-plantar erythrodysesthesia syndrome	13 (22)	1 (2)
Rash	13 (22)	0
Dysgeusia	12 (20)	0
Hoarseness	12 (20)	0

*AE* adverse event

^a^Reported in ≥20 % of patients

Four treatment-related AEs led to permanent axitinib discontinuation: headache (Grade 1), cerebrovascular accident (Grade 4), proteinuria (Grade 2), and weakness (Grade unknown). AEs led to axitinib dose reductions in 25 (42 %) patients. Diarrhea (10 %), fatigue (10 %), hypertension (7 %), and palmar-plantar erythrodysesthesia (5 %) were most frequently associated with dose reductions. Hypertension was managed with antihypertensive medication. No patients discontinued the study because of hypertension; BP elevations were generally resolved by the next assessment.

### Pharmacokinetic/pharmacodynamic analyses

**Fig. 2:**
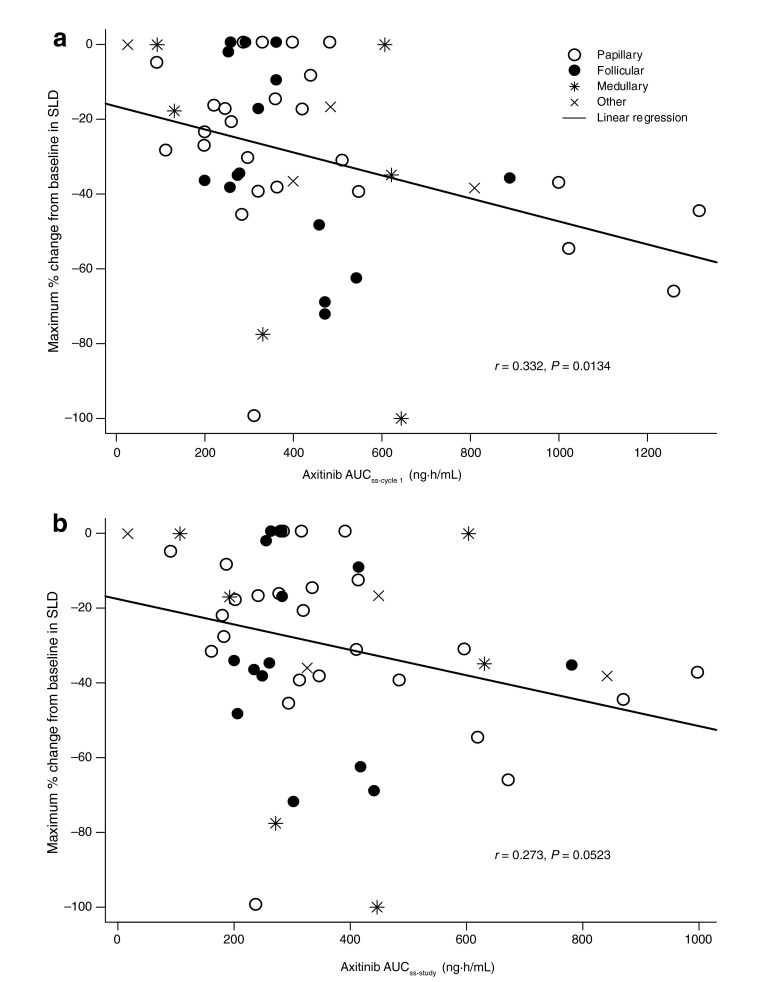
Maximum percent change from baseline observed at any point during the study in the sum of longest diameter (SLD) of target lesions relative to axitinib area under the plasma concentration–time curve at steady-state (AUC_ss_) during **a** cycle 1 (AUC_ss-cycle1_) and **b** the entire time on study (AUC_ss-study_); although no patients in the study had a RECIST-defined complete response, two patients had target lesions that became unmeasurable during axitinib treatment (−100 % change)

**Fig. 3:**
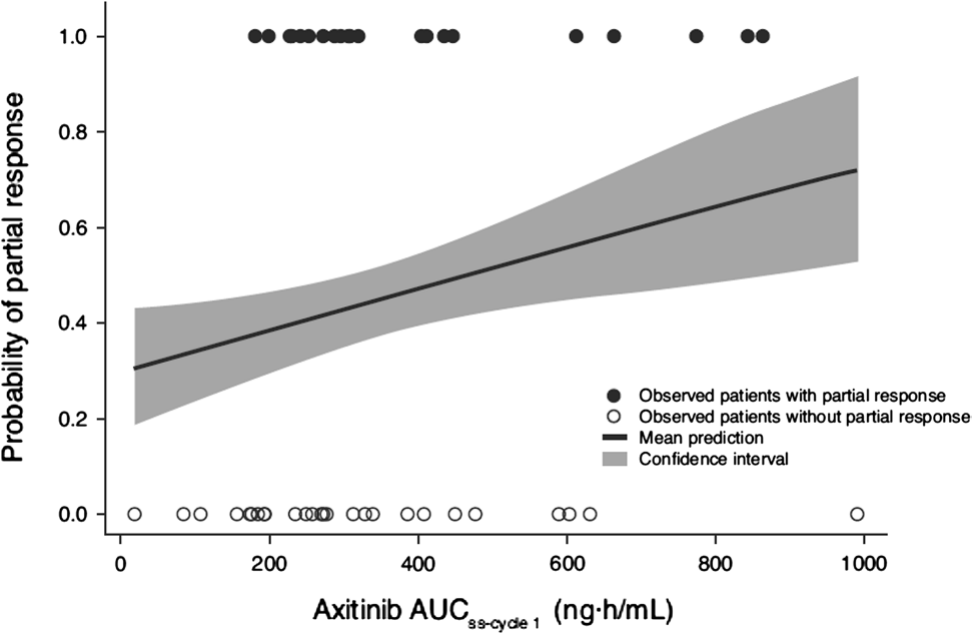
Logistic regression analysis of probability of an investigator-assessed partial response relative to axitinib area under the plasma concentration–time curve at steady-state during cycle 1 (AUC_ss-cycle1_)

In all, 49 of 60 patients had adequate PK data to calculate post hoc AUC_ss_ and were included in the PK/PD analyses. Greater reduction in tumor size (Fig. 2), assessed by maximum percent change from baseline in SLD of target lesions, was seen with increasing axitinib AUC_ss-cycle1_ (*r* = 0.332; *P* = 0.0134) and AUC_ss-study_ (*r* = 0.273; *P* = 0.0523). As a measure of inherent axitinib exposure (i.e., before dose titration that could occur at or beyond 8 weeks), AUC_ss-cycle1_ was used as a measure of drug exposure in individual patients for the remaining PK/PD analyses. Figure 3 provides a comparison of steady-state plasma exposures in patients who had PR (denoted at 1.0 on the *y*-axis) versus those who did not have PR (denoted at zero on the *y*-axis). These raw data were subjected to logistic regression to obtain the overlaid curve, which describes the probability of having PR as a function of axitinib plasma exposure. This analysis indicated that patients with higher axitinib plasma exposure (AUC_ss-cycle1_) had a greater likelihood of obtaining PR.

**Fig. 4:**
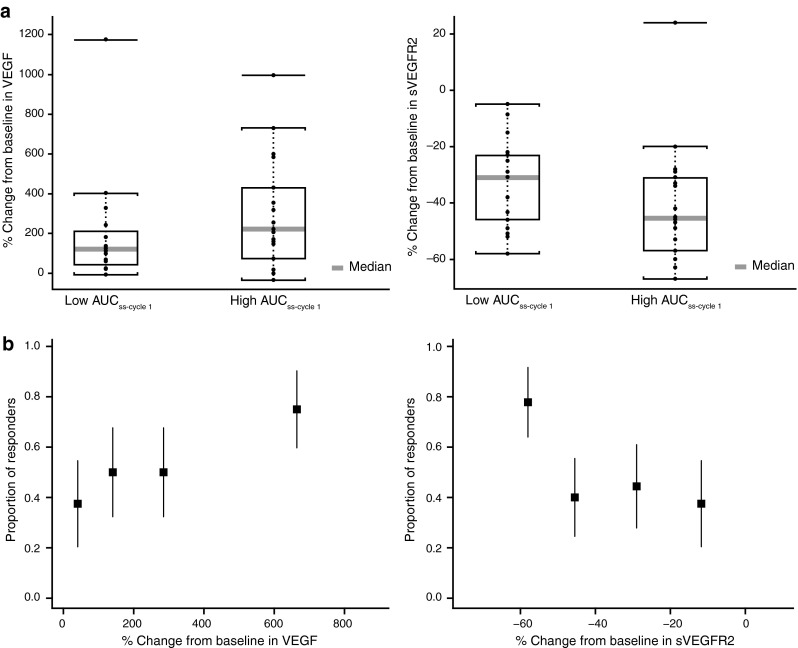
**a** Greatest percent change from baseline per patient at any time during the study in vascular endothelial growth factor (VEGF) and soluble VEGF receptor 2 (sVEGFR2) in patients with high (≥mAUC_ss-cycle1_) and low (<mAUC_ss-cycle1_) exposure to axitinib, and **b** proportion of patients with investigator-assessed partial responses in quartiles grouped according to change from baseline in soluble proteins. mAUC_ss-cycle1_, median area under the plasma concentration–time curve at steady-state during cycle 1

Axitinib was previously reported to lead to a 2.8-fold increase in mean VEGF and 32 % decrease in mean sVEGFR2 concentrations that plateau by week 12 [16]. In the current analysis, patients with exposure to axitinib that was equal to or greater than median AUC_ss-cycle1_ had greater median VEGF increases and sVEGFR2 decreases (Fig. 4a). When grouped into quartiles according to percent change from baseline in VEGF and sVEGFR2 concentrations, higher proportions of responses were observed in patients with the greatest increases from baseline in VEGF and those with the greatest decreases from baseline sVEGFR2 (Fig. 4b). When stratified by the length of time patients received axitinib (≥1 vs. <1 year), no differences were observed in median VEGF increases or sVEGFR2 decreases from baseline (data not shown).

## Discussion

A 38 % ORR and 21-month median duration of response for axitinib 5 mg twice daily is reported in patients with advanced thyroid cancer. Although the sample size was too small for definitive assessment, follicular histology appeared to be most responsive to axitinib (Table 1). Stable disease lasting ≥16 weeks, as defined by the protocol, occurred in an additional 18 (30 %) patients; however, 17 of those patients had stable disease lasting ≥30 weeks. These translated into 15-month median PFS and 35-month median OS. Results from another Phase II trial of axitinib in patients (*N* = 52) with ^131^I-refractory advanced thyroid cancer demonstrated a comparable 35 % ORR, 16.1-month median PFS, and 27.2-month median OS [15]. These two trials consistently demonstrated that axitinib has activity in the treatment of advanced thyroid cancer.

Since documented disease progression was not required prior to enrollment in this trial, PFS and OS data may be difficult to interpret in light of the natural history of metastatic thyroid cancer. Several randomized placebo-controlled Phase III trials evaluating VEGFR TKIs have now been conducted in patients with advanced thyroid cancer [11, 17, 24]. Patients receiving placebo in those trials had median PFS of 4 months [24] versus 19.3 months [17] for medullary thyroid cancer (MTC) with versus without progressive disease at study entry. Likewise, patients with progressive differentiated thyroid cancer (DTC) who received placebo had median PFS of 5.8 months [11]. In a randomized placebo-controlled Phase II trial, 16 (22 %) of 73 patients with DTC who received placebo had stable disease for a period of 12 months [18]. Whereas median OS has not been reported from these placebo-controlled trials, the survival analyses may be confounded by crossover to active treatment. Several single-arm Phase II trials have evaluated VEGFR TKIs [6–10, 12–14, 19–21, 25] for the treatment of advanced thyroid cancer (summarized in Online Resource 1). Only a few trials have reported median OS in patients treated with sorafenib: 23 and 38 months in non-progressive papillary thyroid cancer that was chemotherapy-naïve or previously treated with chemotherapy [7], respectively, and 35 months in progressive DTC [10].

The National Comprehensive Cancer Network 2013 Clinical Practice Guidelines [34] suggest consideration of systemic therapy with small molecule kinase inhibitors for clinically progressive or symptomatic metastatic ^131^I-refractory DTC. Additionally, European Society for Medical Oncology 2012 Clinical Practice Guidelines [35] recommend enrollment in clinical trials with TKIs for patients with metastatic DTC. Results from the current study suggest that axitinib may be a potential treatment option for ^131^I-refractory advanced DTC that is progressive or symptomatic. In long-term follow-up, the AE profile for axitinib was similar to previously reported results [16], thus confirming axitinib is well tolerated, with manageable toxicities.

The results reported here suggest there is an increased likelihood of an objective response in patients with the greatest VEGF increases and sVEGFR2 decreases; however, due to the considerable overlap observed in proportions of responders and change in VEGF/sVEGFR2 quartiles, these may not be optimal biomarkers to predict response to axitinib. A previous study also suggested that changes in sVEGFR2 levels after initiation of motesanib might predict response in patients with metastatic DTC or MTC [36]. In the current study, patients with higher axitinib AUC_ss-cycle1_, a measure of inherent axitinib exposure, had greater reduction in tumor size, probability of PR, and greater median VEGF increases and sVEGFR decreases from baseline. In patients with metastatic RCC, greater axitinib exposure has also been associated with a higher ORR and longer PFS and OS [37]. Other investigators have conducted PK/PD analyses in patients with thyroid cancer. For example, motesanib AUC_ss_ was a better predictor of response than dose [38]. Also, maximum pazopanib plasma concentration during the first treatment cycle correlated with maximum change in tumor size and was significantly higher in patients who achieved responses [19].

Limitations of the present trial included the number of patients with indeterminate or missing response assessments (*n* = 14), and the lack of control group and independent radiology review of response. Additionally, results for the 13 patients who continued axitinib for long periods of time in the extension study were based on data collected from this original trial. The extension study is ongoing and as of the cutoff date of June 1, 2012, duration of treatment ranged from 2.6 to 7.5 years in eight patients no longer receiving axitinib and from 6.2 to 7.4 years in five patients still receiving axitinib. Although AE data for long-term axitinib in the extension study were not yet available, five patients still on treatment were receiving total daily axitinib doses of 6–12 mg (data on file; Pfizer Inc, New York, NY, USA).

In conclusion, axitinib appears to be active in various histologic subtypes of advanced thyroid cancer, as evidenced by a high ORR and long median duration of response, PFS, and OS. A limited number of patients with anaplastic histology (*n* = 2) were enrolled, making definitive conclusions in this subtype impossible. Axitinib also demonstrated a generally favorable safety profile. Moreover, greater axitinib exposure was associated with favorable differences in biomarkers and reduction in tumor size. PK/PD analyses suggest that patients with higher axitinib exposure were more likely to achieve PR, thereby providing further rationale for dose increases in patients who tolerate the starting dose of 5 mg twice daily without elevated BP. Overall, these data suggest axitinib may be useful in the treatment of ^131^I-refractory advanced thyroid cancer, and individualized dose titration based on tolerability and BP assessment is warranted. Larger randomized trials are necessary to confirm the role of axitinib for the treatment of advanced thyroid cancer.

## Electronic supplementary material

Below is the link to the electronic supplementary material.

Supplementary material 1 (DOCX 19 kb)
