# Supplementary material for: A Phase II trial of axitinib in patients with various histologic subtypes of advanced thyroid cancer: long-term outcomes and pharmacokinetic/pharmacodynamic analyses
Source: Cancer Chemother Pharmacol. 2014 Oct 15;74(6):1261–70. doi: 10.1007/s00280-014-2604-8 (PMC4236619; doi:10.1007/s00280-014-2604-8)
Supplement: Supplementary file 1 — Supplementary material 1 (DOCX 19 kb) [file 280_2014_2604_MOESM1_ESM.docx]

Online Resource 1

**Efficacy of select VEGFR TKIs from Phase II trials in patients with advanced thyroid cancer**

| **VEGFR TKI Author** | **Key disease characteristics** | **Thyroid Cancer**  **Histology, subtype (*n*)** | **ORR, *n* (%) [95 % CI]** | **Median PFS, mo (95 % CI)** |
| --- | --- | --- | --- | --- |
| **Axitinib** | | | | |
| Current study  Locati [15] | Refractory or not amenable to RAI  RECIST-defined target lesion  No prior VEGFR-targeted therapy  Refractory or not amenable to RAI  RECIST-defined PD within 12 mo and target lesion  No prior VEGFR-targeted therapy | DTC (45); MTC (11); anaplastic (2); other (2)  DTC (46); MTC (6) | 23 (38) [26–52]^a^  18 (35) [22–49]^a^ | 15 (10–20)^a^  16.1 (14.8–21.6)^a^ |
| **Lenvatinib** | | | | |
| Schlumberger [25] | RECIST-defined PD within 12 mo  Prior VEGFR-targeted therapy allowed | MTC (59) | 21 (36) [29–49]^b^  29 (49) [32–62]^a^ | 9 (7-NR)^b^ |
| **Motesanib** | | | | |
| Schlumberger [20]    Sherman [21] | RECIST-defined PD within 6 mo or symptomatic disease  RECIST-defined target lesion  Refractory to RAI  RECIST-defined PD within 6 mo and target lesion | MTC (91)  DTC (93) | 2 (2) [0.3–8]^b^  13 (14) [8–23]^b^ | 11 (10–13)^b^  9 (7–12)^b^ |
| **Pazopanib** | | | | |
| Bible [19] | Refractory to RAI  RECIST-defined PD within 6 mo | DTC (37) | 18 (49) [35–68]^a^ | 12 (NR)^a^ |
| **Sorafenib** | | | | |
| Gupta-Abramson [6]    Kloos [7]  Ahmed [8]    Lam [9] | Refractory to RAI  RECIST-defined PD within 12 mo and measurable disease  1 prior kinase inhibitor allowed  Measurable disease  PD not suitable for RAI  RECIST-defined measurable disease  Measurable disease | DTC (27); MTC (1); Other (2)  PTC (41)  DTC (19); MTC (15)  MTC (16) | 7 (23) [10–42]^a^  6 (15) [6–29]^a^  7 (21) [8–40]^a,c^  1 (6) [0.2–30]^a^ | 18 (NR)^a^  15 (10–28)^a^  NR  17.9 (NR)^a^ |
| **Sunitinib** | | | | |
| Carr [12] | Refractory to RAI  RECIST-defined measurable disease  Prior VEGFR-targeted therapy allowed | DTC (28); MTC (7) | 11 (31) [16–47]^a^ | 13 (9–not reached)^a,d^ |
| Cohen [13] | Refractory or not amenable to RAI  PD within 6 mo | DTC (38) | 7 (18) [NR] | NR |
| De Souza [14] | PD within 6 mo | MTC (24) | 8 (33) [16–55]^a^ | 11 (0.5–36)^a^ |

*CI* confidence interval, *DTC* differentiated thyroid cancer, *MTC* medullary thyroid cancer, *NR* not reported, *ORR* objective response rate, *PD* progressive disease, *PFS* progression-free survival, *PTC* papillary thyroid cancer, *RAI* radioactive iodine, *RECIST* Response Evaluation Criteria in Solid Tumors, *TKI* tyrosine kinase inhibitor, *VEGFR* vascular endothelial growth factor receptor

^a^ Assessed by investigator

^b^ Assessed by independent imaging review

^c^ At 12 months

^d^ Time to progression
